# Xanthene and Xanthone Derivatives as G-Quadruplex Stabilizing Ligands

**DOI:** 10.3390/molecules181113446

**Published:** 2013-10-30

**Authors:** Alessandro Altieri, Antonello Alvino, Stephan Ohnmacht, Giancarlo Ortaggi, Stephen Neidle, Daniele Nocioni, Marco Franceschin, Armandodoriano Bianco

**Affiliations:** 1 Dipartimento di Chimica, Università di Roma “La Sapienza”, Piazzale Aldo Moro 5, Roma 00185, Italy; E-Mails: antonello.alvino@uniroma1.it (A.A.); giancarlo.ortaggi@uniroma1.it (G.O.); daniele.nocioni@tiscali.it (D.N.); marco.franceschin@uniroma1.it (M.F.); 2 The UCL School of Pharmacy, University College London, 29-39 Brunswick Square, London WC1N 1AX, UK; E-Mails: s.ohnmacht@ucl.ac.uk (S.O.); s.neidle@ucl.ac.uk (S.N.)

**Keywords:** G-quadruplex, xanthene, xanthone, ESI-MS, FRET, telomere, c-myc, bcl-2

## Abstract

Following previous studies on anthraquinone and acridine-based G-quadruplex ligands, here we present a study of similar aromatic cores, with the specific aim of increasing G-quadruplex binding and selectivity with respect to duplex DNA. Synthesized compounds include two and three-side chain xanthone and xanthene derivatives, as well as a dimeric “bridged” form. ESI and FRET measurements suggest that all the studied molecules are good G-quadruplex ligands, both at telomeres and on G-quadruplex forming sequences of oncogene promoters. The dimeric compound and the three-side chain xanthone derivative have been shown to represent the best compounds emerging from the different series of ligands presented here, having also high selectivity for G-quadruplex structures with respect to duplex DNA. Molecular modeling simulations are in broad agreement with the experimental data.

## 1. Introduction

**Figure 1:**
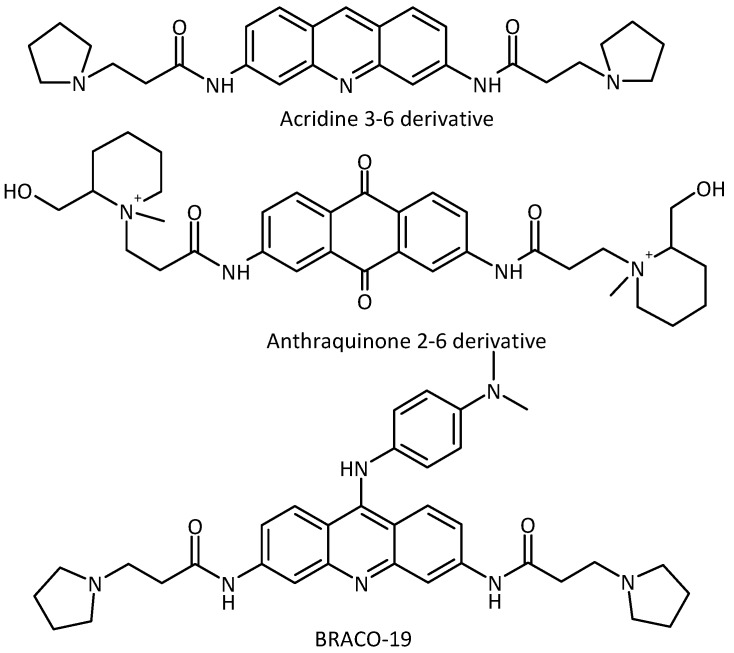
Examples of anthraquinone and acridine derivatives known as DNA G-quadruplex DNA G-quadruplex ligands.

The first G-quadruplex binding ligand having an effect on telomerase activity was discovered in 1997 as a result of the collaboration between the Neidle and Hurley groups [1,2]. Particular attention has subsequently been devoted to the stabilization of these structures involved in key biological processes by small organic molecules, [3,4,5] especially in telomere regions [6,7,8]. However, the first anthraquinone ligands show high levels of non-specific cytotoxicity, possibly due to redox cycling [9,10]. Subsequently, in order to solve this biological problem, a new ligand core was studied: the acridine moiety [11]. The acridine core was chosen in part for its similarity with anthraquinones [12]. A small library of 3,6-disubstituted acridines was synthesised: this showed substantial improvement with respect to the previous series of anthraquinones, *i.e.*, micromolar telomerase inhibition and lower cytotoxicity. Neidle and co-workers also increased the number of side chains on the acridine core, synthesising a library of 3,6,9-trisubstituted acridines [13]. The lead compound, BRACO-19 (Figure 1) is one of the most studied G-quadruplex binding ligands to date, showing significant telomerase inhibitory activity (telEC_50_ = 6.3 μM) [11].

**Figure 2:**
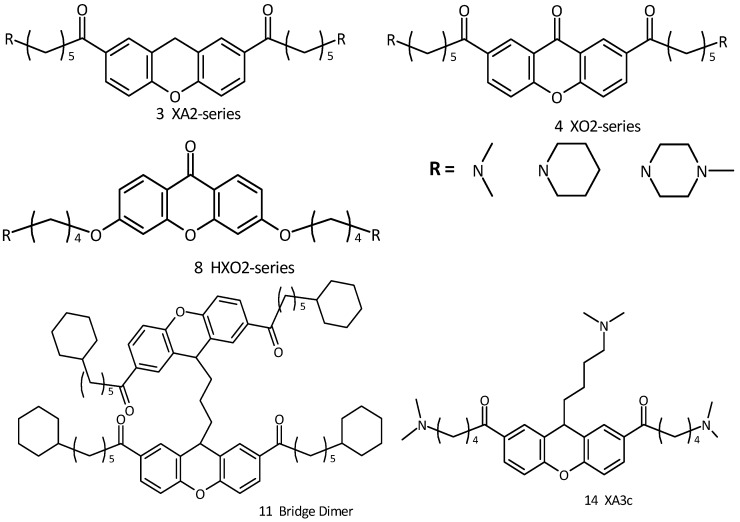
Xanthene and xanthone derivatives as G-quadruplex stabilizing ligands.

In this context we wished to study aromatic cores similar to those previously described so as to induce quadruplex structures, and also possibly reducing the problems related to their unspecific cytotoxicity. In particular, our efforts have been directed towards synthetic targets represented by 3,6 and 2,7-disubstituted xanthene and xanthone derivatives (Figure 2). This core, widely found in Nature, represents a unique class of biologically active compounds possessing numerous bioactive capabilities such as antioxidant properties [14]. These molecules constitute a restricted group of plant polyphenols, biosynthetically related to flavonoids [15]. We developed this core with the specific aim of increasing telomerase activity by rational design [16,17,18]. Molecular modeling studies predict that the xanthene moiety is at least comparable to the anthraquinone and acridine moiety in terms of G-quadruplex binding affinity [19,20,21]. It is an inherently planar chromophore and also contains a heterocyclic oxygen atom which could confer water solubility compared to the analogous anthraquinone core, which is completely insoluble in water.

## 2. Results and Discussion

### 2.1. Design and Molecular Docking

We used a virtual screening computer-based technique for identifying promising compounds to bind to a known G-quadruplex structure. Here we have adopted a docking screening method available in the AutoDock suite of programs. This is a software suite for predicting the optimal bound conformations of ligands to macromolecules [22,23,24]. Its use has been supported by a review by Trent and co-workers [25] showing that AutoDock optimally balances docking accuracy and ranking. This application and also the more general problem of modelling individual quadruplex structures have some similarities to those of other nucleic acid modelling. There is also the added complexity of the central ion channel and the intrinsic flexibility of the telomeric quadruplex itself. More recently, however, enhancements in the performance of AutoDock combined with the increased availability of high speed computers and computer clusters have allowed much larger computational experiments to be undertaken, where entire compound libraries are screened against pharmaceutically-relevant targets. We obtained the initial coordinates for the docking from the Protein Data Bank coordinates of the crystal structure of the parallel 22-mer telomeric G-quadruplex (PDB ID: 1KF1) which shows a single topology, the parallel fold [26].

The corresponding intermolecular energy values were used to calculate the average binding energies (and the relative standard deviations), reported in Table 1. Repeated experiments show good reproducibility, suggesting that the number of structures generated was sufficient to be statistically significant. The binding poses calculated for these compounds were then visually inspected to discard all the ligands which were not able to form hydrogen bonds with any of the guanine bases and/or to establish an electrostatic interaction with the backbone phosphate groups.

**Table 1:** Docking binding energies (kcal/mol) for different compounds with 1KF1. The rmsd values for the whole complex are <4 Å.

Compound	Binding Energy
XA2dma **3a**	−4.6
XA2pip **3b**	−4.4
XA2mpz **3c**	−4.5
XO2dma **4a**	−5.3
XO2pip **4b**	−5.0
XO2mpz **4c**	−5.4
HXO2dma **8a**	−5.8
XO2pip **8b**	−5.5
XO2mpz **8c**	−5.5
Bridge Dimer **11**	−7.0
XA3c **14**	−6.4
*Anthraquinone 2,6 derivative*	−4.1
*Acridine 2-6 derivative*	−4.6
*BRACO-19*	−7.0

There are several limitations to this methodology, mainly related to the inability of AUTODOCK and other docking programs to fully account for the non-rigidity of the quadruplex DNA structure and the likely significant polarisation effect of positive charges on the ligand molecule. Several attempts have been made to try to solve these problems, and it is clear that the calculated binding energies must not be considered as absolute values, but rather as indicative of relative ranking, which can be most useful with a series of homologous molecules having similar chemical structure [27]. However, these docking studies are in good qualitative agreement with the “threading intercalation” model proposed for this type of molecule by Hurley and coworkers, in which the drug is stacked on the terminal G-tetrad, stabilized by π-π interactions with the central aromatic core, while the side chains interact with the G-quadruplex grooves [28].

**Figure 3:**
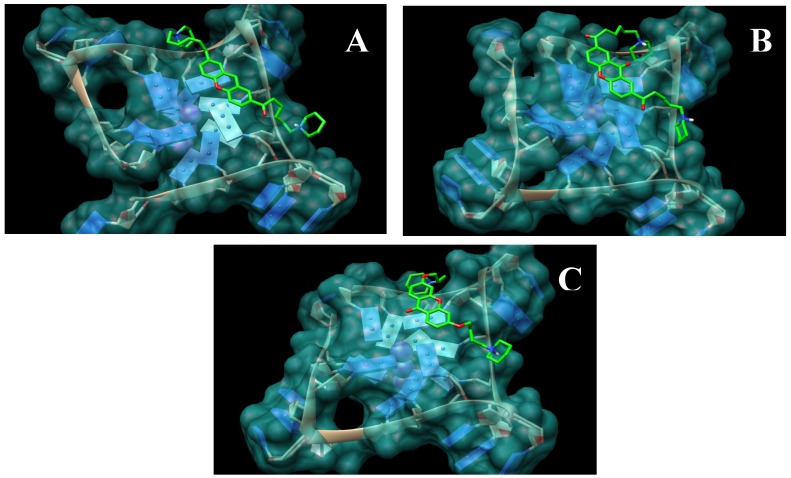
Models obtained for the complexes between xanthene and xanthone derivatives (atom-type coloured) and the telomeric G-quadruplex (blue) (**A**) XA2pip **3b**. (**B**) XO2pip **4b**. (**C**) HXO2pip **8b**.

The first series of xanthene-based G-quadruplex binding ligands XA2 (dma, pip and mpz; **3a**,**b** and **c**) and XO2 (dma, pip and mpz, **4a**,**b** and **c**) have been the starting point of this work. Docking experiments were performed with the xanthene core on a G-quadruplex monomeric structure. These studies have shown good superimposition of the designed ligands with the terminal G-tetrad of the G-quadruplex (Figure 3), similar to that shown by anthraquinone and acridine derivatives. The binding energies calculated for molecules with a xanthenic core are in overall accord with those calculated for known ligands (Table 1).

The second class of derivatives HXO2 dma, pip and mpz (**8a**,**b** and **c**) has been designed with the aim of improving the binding ability of previous models. We wished to obtain a compound with a greater number of oxygen atoms necessary to ensure good aqueous solubility. In view of the results from the docking simulations, we considered it appropriate to functionalize the planar structure of the xanthone in positions 2 and 7 so that the side-chains are oriented in the correct direction to be able to interact with two of the four adjacent quadruplex grooves generated by one of the loops. Moreover the carbonyl oxygen atom of the xanthone is directed away from the loop, preventing the xanthone core position becoming more decentralized and near the loop. The xanthone core appears displaced toward the central ion channel and this causes a further distancing of the side-chains from the grooves. Most of the structures obtained for the compounds of the first series on the 3’ G-tetrad face show the ligand molecule in a position analogous to the one represented in Figure 3(B): one side-chain is well fitted into one of the four grooves (regardless of which one), while the other is more flexible, since it cannot reach another groove. Molecular docking studies show how the second generation of ligands has an inverted behaviour compared to the earlier compounds. It is notable that the carbonyl oxygen atom of the xanthone is oriented towards the centre of the structure, where it is stabilized by a K^+^ ion situated between the tetrads [29]. Therefore, the side chains are closer to the grooves and they have to be shorter than those linked in position 3 and 9: only four carbon atoms compared to six for the first series are necessary.

**Figure 4:**
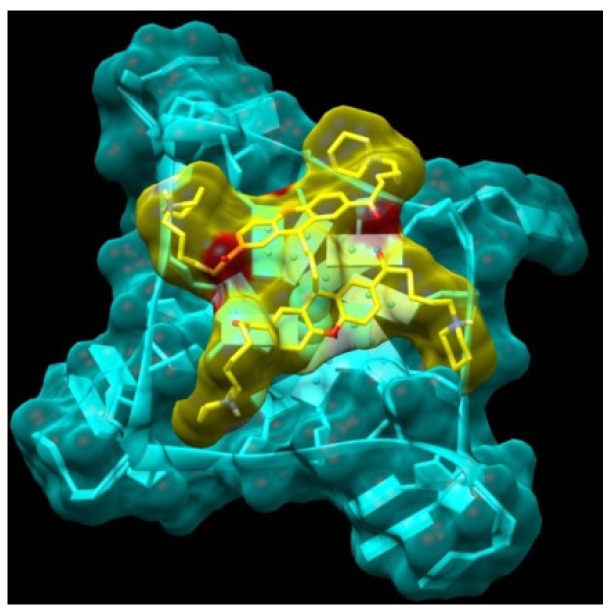
Model obtained for the complex between the Bridge Dimer (**11**, yellow atom-type) and the monomeric G-quadruplex (light blue).

Small aromatic cores such as xanthene, when functionalized with hydrophilic and positively charged chains, can intercalate and interact with double-stranded DNA, as demonstrated in the following sections. For this reason, we attempted to design more selective quadruplex ligands. As a first approach, we expanded the surface of the aromatic core, creating a bridge between two xanthene groups. Since the first series of compounds are predicted to be able to interact with two of the four quadruplex grooves through their two side-chains, our aim was to add a small bridge that would enable the new molecule dimer bridge (**11**) to interact with all four quadruplex grooves, with a consequent improvement in selectivity and stability. The two monomer units can rotate independently and adapt to terminal tetrad binding site, with distinct orientations: a possible model of interaction is shown in Figure 4.

**Figure 5:**
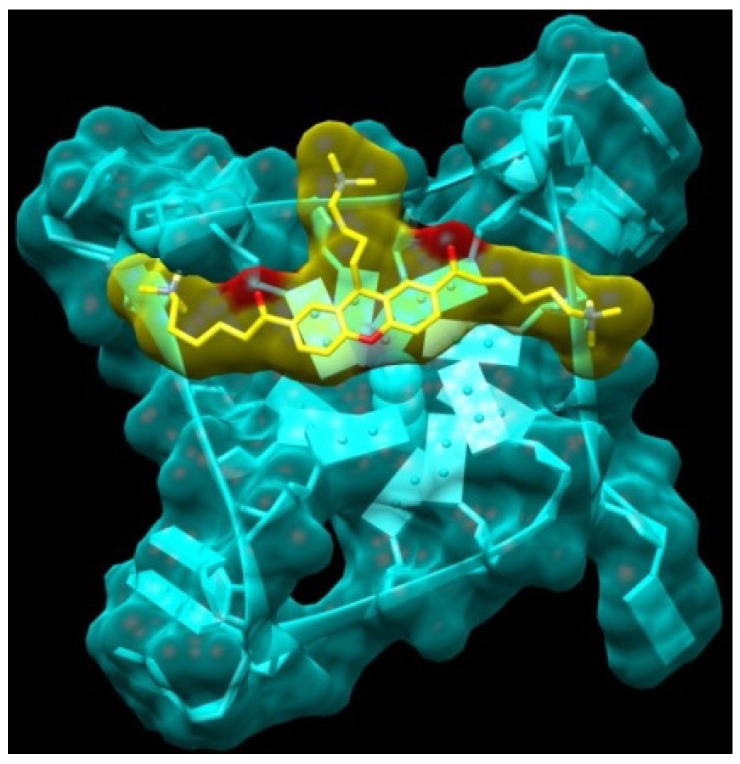
Model obtained for the complex between the XA3c (14), yellow atom-type) and the monomeric G-quadruplex (light blue).

In view of the findings from earlier studies that the introduction of an appropriate third substituent arm enhances selectivity (for instance trisubstituted triazines, porphyrins, and acridines such as BRACO-19), we considered it appropriate to introduce a third chain on the xanthone core, using the knowledge already acquired from the two-chain templates [13,30]. The calculated binding energy of compound XA3c (**14**) looks promising. The interaction of this compound is predicted with the telomeric quadruplex to show the positively charged side chains in position 3 and 6 each extending into a wide groove, while the side chain in position 9 inserts into a narrow hydrophobic pocket (Figure 5).

### 2.2. Synthesis

**Scheme 1:**
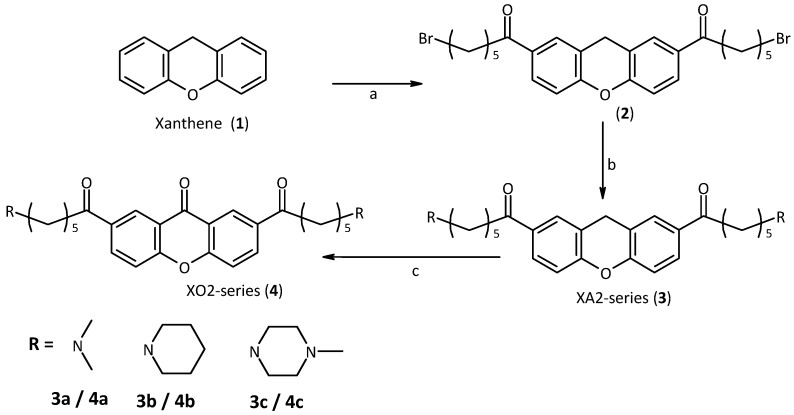
Synthesis of the first series of xanthene and xanthone derivatives.

Following the molecular modelling studies, the aim of our work became the synthesis of xanthene and xanthone derivatives appropriately modified in order to improve aqueous solubility and ability to interact with the target quadruplex. We started from xanthene itself, which is commercially available. The first step in the strategy was to introduce the side-chains by Friedel-Crafts acylation. In this way, the 2,7-positions were functionalized. We employed chains of suitable length which are also very flexible, to be in optimal contact with the quadruplex backbones and grooves [31]. Since these chains terminate with a bromine atom, suitable for the subsequent nucleophilic substitution, using different amines, similar to those used for this purpose in the literature, we obtained a small library of compounds such as XA2 dma, pip and mpz (**3a**,**b** and **c**, Scheme 1) [32,33]. The secondary amines we used become tertiary after substitution and are charged under physiological conditions. To increase the planarity of the molecule, we decided to oxidize the methylene group of the xanthene to a carbonyl group with Jones reactant, obtaining the corresponding xanthone derivatives XO2 dma, pip and mpz (**4a**,**b** and **c**, Scheme 1).

*Reagents and Conditions*: (**a**) 6-Bromohexanoyl chloride, AlCl_3_, DCM, 0 °C Ar 5 h, 86% yield; (**b**) HR, DCM, r.t. 12 h, 93%–96% yield; (**c**) Cr_2_O_3_, H_2_SO_4_, acetone, 0 °C, 5 h, 93%–96% yield.

**Scheme 2:**
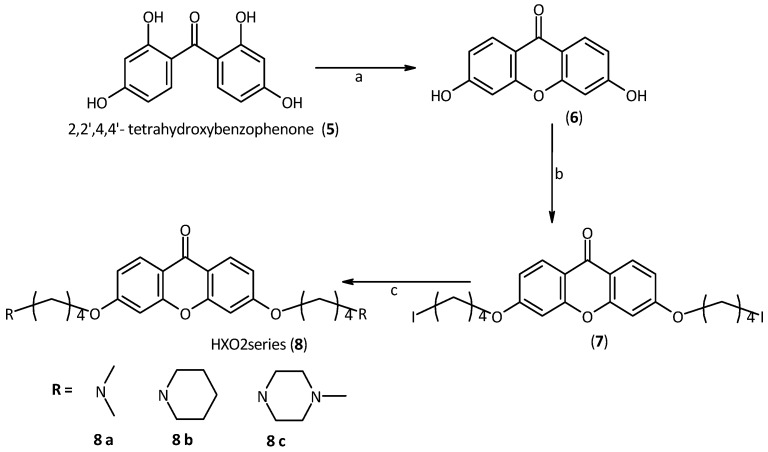
Synthesis of the second series of xanthone derivatives.

Regarding the synthesis of the second generation of derivatives (HXO2 dma, pip and mpz, **8a**,**b** and **c**) we had to synthesize the xanthone core, because the 3 or 6 positions of this core are deactivated. The starting-point was 2,2',4,4'-tetrahydroxybenzophenone, which was dehydrated by generating a xanthone core with two hydroxyl groups in the desired 2 and 7 positions. This reaction is quantitative and occurs only under high temperature and pressure conditions. To carry out this dehydration, the starting material was suspended in a sealed steel tube half-filled with water. At 250 °C, the water evaporation generates the necessary pressure (about 50 atm) to promote the reaction [34] Then, side chains of suitable length were introduced, using 1,4-diidodoproane. As in the previous series of compounds, the second halogen was replaced with the desired amine. Piperidine, methylpiperazine and dimethylamine were used as amines to obtain a small library of derivatives, Scheme 2.

*Reagents and Conditions*: (**a**) H_2_O, 210 °C, 50 atm, 4 h, 99% yield; (**b**) I(CH_2_)_4_I, K_2_CO_3_, DCM, 50 °C, 12 h, 91% yield; (**c**) HR, DCM, r.t. 12 h 91%–97% yield.

**Scheme 3:**
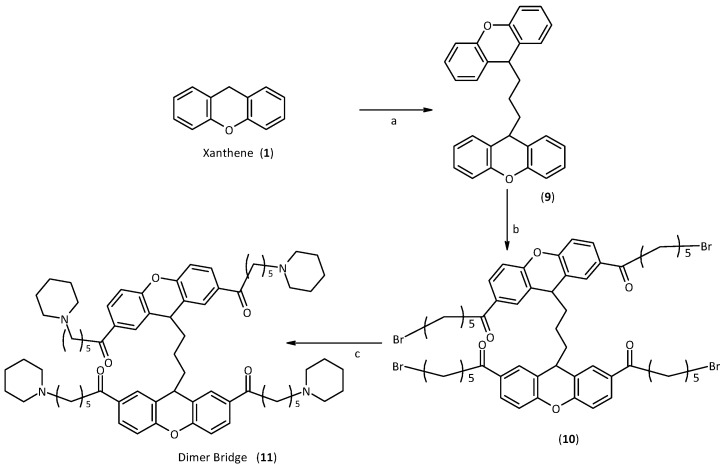
Synthesis of the Dimer Bridge.

The first step in the synthesis of the bridge dimer **11** was the creation of the bridge between the two xanthene moieties. This reaction first requires generation *in situ* of the corresponding carbanion, and then the addition of a stoichiometric amount of 1,3-diiodopropane. At this point, the synthesis was performed as for the first generation of xanthene. Therefore, a Friedel Craft acylation was carried out on the two positions 2 and 7, and finally the subsequent nucleophilic substitution with the amine, Scheme 3.

*Reagents and Conditions*: (**a**) BuLi (6 mmol), I(CH_2_)_3_I (1 mmol), THF, Ar, 0 °C 2 h, 42% yield; **(b**) 6-bromohexanoyl chloride (4 mmol), AlCl_3_, DCM, 0 °C Ar 5 h, 84% yield; (**c**) piperidine, DCM, r.t. 12 h, 90% yield.

**Scheme 4:**
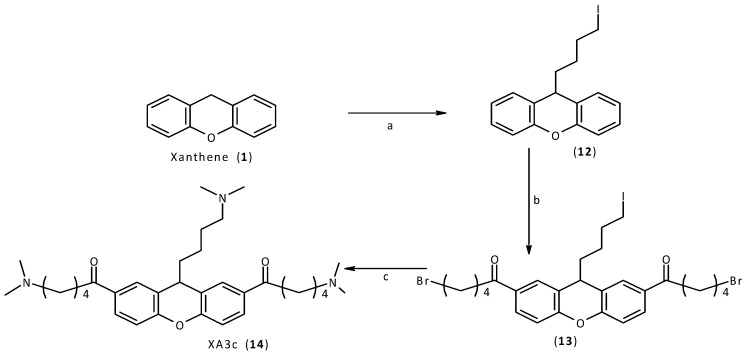
Synthesis of XA3c (**14**).

Finally, a similar procedure was applied for the synthesis of the final compound, XA3c (14). Using an excess of 1,3-diiodopropane, it was possible to introduce only one chain on the xanthene core. The acylation and the following replacement were performed as already described. In the final step of this synthetic procedure, the secondary amine simultaneously displaces both bromine and iodine atoms of the intermediate compound, to give the final desired compound XA3c (14, Scheme 4).

*Reagents and Conditions*: (**a**) BuLi (5 mmol), I(CH_2_)_4_I (4 mmol), THF, Ar, 0 °C 2 h, 48% yield; (**b**) 6-bromohexanoyl chloride (4 mmol), AlCl_3_, DCM, 0 °C Ar 5 h, 84% yield; (**c**) HN(CH_3_)_2_, DCM, r.t. 12 h, 76% yield.

### 2.3. Studies of Ligands Interactions with G-Quadruplex and Duplex DNA by ESI-MS Experiments

Electrospray ionization mass spectrometry (ESI-MS) is a powerful tool for studying biomolecular structures and non-covalent interactions. This technique allows the transfer of non-covalently bound complexes into the gas phase without the disruption of the complex itself and therefore the determination of the stoichiometry and, in particularly favourable cases, modes and energies of interaction. For the analysis of noncovalent complexes between nucleic acids and small molecules, De Pauw and co-workers used ESI-MS to study the interaction of double-stranded DNA with two classes of antitumor drugs: intercalators (ethidium bromide, amsacrine and ascididermin) and minor groove binders (distamycin A, Hoechst 33258, netropsin, berenil and DAPI) [35,36]. They also evaluated the DNA affinities of the minor groove binders, by quantifying the equilibrium association constants of the observed complexes and they demonstrated consistency of these values with those obtained from other traditional techniques. In the past few years, ESI-MS has been used in the study of nonconventional DNA structures, including DNA triplexes and especially G-quadruplexes [37]. It has been successfully applied to the study of the binding of G-quadruplex ligands to their target sequences in order to determine stoichiometry and relative binding affinities of such complexes [38]. Quantitative analysis of binding affinities with quadruplex DNA structures is possible, because the association constants can be calculated directly from the relative intensities of the corresponding peaks found in the mass spectra, with the assumption that the relative intensities in the spectrum are proportional to the relative concentrations in the injected solution.

For this study, we have chosen two oligonucleotides that can form different G-quadruplex and duplex structures: HTelo21 (5'-GGGTTAGGGTTAGGGTTAGGG-3') which comprises human telomeric repeats and is able to fold into a monomeric G-quadruplex structure, as characterized by X-ray crystallography and NMR. The association constants have been calculated directly from the corresponding peaks found in the mass spectra, since the relative intensities in the spectrum are proportional to the relative concentrations in the injected solution, as previously reported [39,40].

In order to evaluate ligand selectivity for quadruplex over duplex DNA we have studied its affinity for a self-complementary dodecamer: DK66 (5'-CGCGAATTCGCG-3'), one of the duplex DNA models most reported in the literature. The evaluation of the binding constants coming from the collected data demonstrates that all the molecules examined are good telomeric G-quadruplex ligands, able to form both 1:1 and 2:1 drug-DNA complexes. This is in agreement with the terminal stacking mode of interaction between ligands and G-quadruplex DNA, confirmed by the molecular modelling studies reported above (Figure 3), and with the existence of two binding sites, corresponding to the two terminal tetrads, resulting in two different binding constants (K_1_ and K_2_). The synthesized ligands show similar K_1_ values, with log K_1_ = 4.7 ÷ 4.0 for the xanthene derivatives, with XA2pip (**3b**) showing the highest value of K_1_ with log K_1_ = 4.7. In the examination of K_2_ values XA2dma (**3a**), XA2pip (**3b**), and XA2mpz (**3c**) show similar values of K_2_ with log K_2_ = 4.2, 4.6, and 3.9, respectively (Table 2).

**Table 2:** K_1_ and K_2_ values calculated on a logarithmic scale and values of percentage of bound DNA for experiments performed at 1:1 drug: DNA ratio for HTelo21 (or duplex DNA, DK66) and the indicated compounds with standard deviations reported over at least three independent experiments.

Compound	HTelo-21				DK66		
	Log K_1_	Log K_2_	DNA bound 1:1	DNA bound 1:2	Log K_1_	Log K_2_	DNA bound 1:4
XA2dma (**3a**)	4.6 ± 0.1	4.2 ± 0.1	23 ± 3	40 ± 4	3.4 ± 0.2	1.9 ± 0.2	9 ± 5
XA2pip (**3b**)	4.7 ± 0.1	4.6 ± 0.1	24 ± 3	41 ± 5	3.8 ± 0.2	2.1 ± 0.3	11 ± 5
XA2mpz (**3c**)	4.4 ± 0.1	3.9 ± 0.2	21 ± 4	39 ± 5	3.7 ± 0.1	2.0 ± 0.2	11 ± 4
XO2dma (**4a**)	4.0 ± 0.1	3.8 ± 0.1	17 ± 4	32 ± 4	3.3 ± 0.2	1.9 ± 0.3	9 ± 5
XO2pip (**4b**)	4.4 ± 0.1	4.2± 0.2	20 ± 3	39 ± 5	3.7 ± 0.1	2.5 ± 0.2	11 ± 6
XO2mpz (**4c**)	4.1 ± 0.1	3.7 ± 0.2	17 ± 4	33 ± 5	1.8 ± 0.2	1.1 ± 0.3	6 ± 5
HXO2dma (**8a**)	4.7 ± 0.1	3.6 ± 0.1	23 ± 5	35 ± 5	3.6 ± 0.1	3.4 ± 0.1	14 ± 5
HXO2pip (**8b**)	4.7 ± 0.1	4.6 ± 0.1	25 ± 3	41 ± 5	3.4 ± 0.1	‒	12 ± 5
HXO2mpz (**8c**)	4.3 ± 0.1	3.7 ± 0.2	21 ± 5	33 ± 4	3.5 ± 0.1	1.1 ± 0.1	13 ± 5
Bridge Dimer (**11**)	5.8 ± 0.1	5.6 ± 0.2	47 ± 2	57 ± 2	3.9 ± 0.1	3.3 ± 0.1	16 ± 2
XA3c (**14**)	5.2 ± 0.4	4.9 ± 0.1	41 ± 3	50 ± 2	3.6 ± 0.3	2.9 ± 0.2	13 ± 6

Contrary to what expected, the oxidized compounds XO2pip (**4b**), XO2dma (**4a**) and XO2mpz (**4c**) have quadruplex association constants in line with the respective xanthene derivatives. Although their capacity to interact with the terminal tetrads is superior due to their greater planarity, this characteristic, due to self-aggregation phenomena, limits their ability to interact with the desired target. This was also confirmed by their lower solubility in common organic solvents where they are in the form of neutral amines, and also in water where they in the form of the corresponding hydrochloride.

We therefore considered appropriate to continue to develop this core, in order to improve the ability to interact with the terminal G-tetrad. Therefore we have designed and subsequently synthesized the second series of xanthenes, as described above. The data show that also in this instance the ligand functionalized with piperidine has optimal properties. The dimethylamino derivative has similar values of K_1_, although the K_2_ value is 10-fold lower. The results show that all xanthene and xanthene compounds do bind G-quadruplex structures. They are also able to form complexes with duplex DNA, although with about 10-fold lower association constants. We believe that the low selectivity for quadruplex structures is due to the extreme flexibility of the side chains that are able to interact with the grooves of both quadruplex and duplex. Another consideration is that the small size of the central core does not optimize the interactions with the aromatic surface of the terminal G-tetrad.

The results obtained for the bridge dimer are particularly interesting. Besides the expected peak of 1:1 for the DNA-ligand complex, we observed a peak of equal intensity but less defined. We believe it is for a 2:1 DNA-ligand complex. The mass/charge (*m/z*) value provides only the stoichiometry. The complex is found in the same range as the previous one (although the weight is almost doubled) as is the charge for the two oligonucleotides appears to be double. Peaks are normally observed with a formal charge of 5 or 4, while in this case we see a complex with double mass but at the same time also the assumed charge is doubled. Unfortunately, the resolution of the ESI-MS could not provide any more detailed information. It is evident from the data reported, that there is an improvement in overall binding to quadruplex DNA, while binding to duplex DNA remains constant, thus improving selectivity.

As for the instance of the three side chain compound XA3c (**14**), the observed binding constants demonstrate that this is an effective G-quadruplex ligand able to form both 1:1 and 2:1 drug-DNA complexes. This is in agreement with the general model for G-quadruplex-ligand interactions characterized by the presence of two binding sites on the external tetrad surfaces of the G-quadruplex structure, even though other explanations are possible for the 2:1 stoichiometry, since the external tetrads of the quadruplex are not identical. The logarithmic values of K_1_ are above 5.8 for HTelo21, while that for K_2_ is 5.6. Comparing K_1_ and K_2_ we can assume that no cooperativity is involved in the binding mechanism since K_2_ values are always lower than those for K_1_. In order to evaluate the selectivity of XA3c (**14**) for quadruplex over duplex DNA we have undertaken a preliminary study of its affinity for a self-complementary dodecamer: DK66. In this case, the spectra acquired at 1:1 ratios do not show any traces of the 1:1 or 2:1 complex peaks, suggesting weaker interaction compared to that for G-quadruplexes. In order to detect a significant peak of the 1:1 drug-DNA complex, this molecule must be present in the sample at a 2:1 ratio, but even at this concentration, there is no evidence of the peak relative to the 2:1 complex; this peak can be appreciated only at higher concentration and at ratios greater than 1:4.

A more reliable analysis of quadruplex *vs*. duplex selectivity has been undertaken by performing competition experiments in the simultaneous presence of the G-quadruplex-forming oligonucleotide HTelo21 and fragments of double-stranded genomic (calf thymus) DNA [40,41]. We chose only one compound (XA3c, **14**), as the best compound emerging from the previous series of measurements, for these competition experiments (see Table 3). The choice of calculating the “amount bound” is because this parameter is relevant to the specific biological activity of these compounds, since it can be considered representative of their capability to sequester telomeric DNA and so inhibit telomerase activity [42,43].

**Table 3:** Competition experiments on HTelo21 oligo. Values of percentage bound DNA as derived from Equation 7 (see Experimental 3.4) for samples containing a fixed amount of both drug and G-quadruplex DNA (5 μM, 1:1 ratio) and different amounts of calf thymus DNA (CT), at the indicated quadruplex/duplex ratios (in phosphate ions). N is the normalized percentage of bound quadruplex, defined as follows: N = (% quadruplex bound in presence of CT)/(% quadruplex bound in absence of CT).

Parameter	[CT] = 0	[HTelo21]:[CT] 1:1	[HTelo21]:[CT] 1:2
Amount bound	47.2 ± 2	41.2 ± 3	34.8 ± 3
N	1	87.3	73.7

ESI-MS experiments were performed on the most promising and representative compounds with two known promoter G-quadruplex forming sequences, of the oncogenes: bcl2 and c-myc [43].

Also in this instance, on the basis of the data reported in Table 4, there is clearly higher activity for dimeric and three side chains derivatives compared to other series of xanthene and xanthone derivatives.

**Table 4:** K_1_ and K_2_ values (reported on a logarithmic scale) and percentage of bound DNA calculated at 1:1 and 2:1 drug/DNA ratio for the indicated oligonucleotides, with standard deviations reported over at least three independent experiments.

Compound	bcl2				myc2345			
	LogK_1_	LogK_2_	DNA bound 1:1	DNA bound 1:2	LogK_1_	LogK_2_	DNA bound 1:1	DNA bound 1:2
XA2pip (**3b**)	3.1 ± 0.1	3.0 ± 0.3	12 ± 3	26 ± 4	4.1 ± 0.1	4.1 ± 0.1	17 ± 4	33 ± 4
XO2pip (**4b**)	2.0 ± 0.3	1.6 ± 0.2	5 ± 2	15 ± 5	2.2 ± 0.1	2.0 ± 0.2	8 ± 2	17 ± 5
XO2pip (**8b**)	3.4 ± 0.1	2.9 ± 0.2	13 ± 4	28 ± 4	3.9 ± 0.1	3.7 ± 0.3	16 ± 4	30 ± 4
Dimer (**11**)	4.1 ± 0.3	3.2 ± 0.3	17 ± 2	32 ± 2	5.0 ± 0.2	5.0 ± 0.2	39 ± 2	48 ± 2
XA3c (**14**)	5.0 ± 0.1	4.8 ± 0.1	40 ± 3	49 ± 3	5.1 ± 0.1	4.7 ± 0.2	40 ± 3	49 ± 3

### 2.4. FRET Assays of Xanthene and Xanthone Derivatives on Quadruplex and Duplex DNA

Fluorescence resonance energy transfer (FRET) measurements were performed to confirm quadruplex binding activity and selectivity with respect to duplex DNA: two suitable fluorescent probes were bound to different quadruplex and duplex forming sequences, resulting in an efficient system to quickly study both quadruplex and duplex thermal stabilization by different ligands [44]. This technique has been applied both to a human telomeric sequence and to two known promoter G-quadruplex-forming sequences from the oncogenes: bcl2 and c-kit1, as well as to duplex DNA (T loop).

Table 5 shows that the new hydrophilic three side-chain xanthene derivative is a potent G-quadruplex ligand. In particular all the xanthene derivatives show good selectivity with respect to duplex DNA in accordance with the previous mass experiments. Nevertheless, since it may be that the duplex model could be too simplified; in this regard further investigations are required.

**Table 5:** ΔT_m_ data at 1 μM ligand concentration (with 60 mM K^+^ at pH 7.4). Esds are ±0.1 °C.

Compound	F21T	BCL2	CKIT1	T Loop
XA2pip (**3b**)	1.3	0.7	1.0	0.4
XO2pip (**4b**)	2.4	0.8	1.9	1.3
HXO2pip (**8b**)	5.1	3.3	4.1	1.2
XA3c (**14**)	11.9	4.8	7.9	2.2

## 3. Experimental

### 3.1. General

All commercial reagents and solvents were purchased from Fluka (Milano, Italy) and Sigma-Aldrich (Milano, Italy), and used without further purification. TLC glass plates (silica gel 60 F254) and silica gel 60 (0.040–0.063 mm) were purchased from Merck. ^1^H and ^13^C-NMR spectra were obtained with Varian Mercury 300 instruments. ESI-MS spectra were recorded on a Micromass Q-TOF MICRO spectrometer.

### 3.2. Molecular Modelling

The crystal structure used was that of the parallel 22-mer telomeric G-quadruplex (PDB ID: 1KF1) Ligand structures were constructed by adopting Avogadro1.0.3 for force field optimization by using the MMFF94 steepest descent algorithm. Docking studies were performed with the AUTODOCK 4.2 program [22,24]. Water molecules were removed from the PDB file, nonpolar hydrogen atom of the telomeric G-quadruplex were added to their corresponding carbon atoms, and partial atomic charges were assigned, by using ADT [45]. The Lamarckian genetic algorithm (LGA) was used to perform docking calculations. A population of random individuals was initially used (population size: 150), with a maximum number of 25,000,000 energy evaluations, a maximum number of generations of 27,000, and a mutation rate of 0.02. 100 independent docking runs were carried out for each ligand. The resulting positions were clustered according to a root-mean-square criterion of 0.5 Å. Docking module was used to calculate the intermolecular (binding) energy, obtained as a sum of electrostatic and van der Waals contributions, between ligand and DNA. The corresponding intermolecular energy values were used to calculate the average binding energies (and the relative standard deviations.

### 3.3. Synthesis

*Bromo-1-[7-(6-bromohexanoyl)-9H-xanthen-2-yl]-hexan-1-one* (**2**): In a two-necked flask xanthene (**1**, 200 mg, 1.10 mmol) and AlCl_3_ (190 mg, 1.41 mmol) were added to anhydrous DCM (2 mL) at 0 °C under Ar. To the solution 6-bromohexanoyl chloride (0.43 mL, 2.80 mmol) in anhydrous DCM (2 mL) was added dropwise. The reaction was then allowed to warm up to room temperature. After 5 h (TLC 30% hexane-CHCl_3_ 6:4), the solution was neutralized at 4 °C with a saturated solution of sodium bicarbonate. The crude product was than extracted with DCM (3 × 50 mL), dried over Na_2_SO_4_ and taken to dryness *in vacuo*. The crude product was purified by flash column chromatography (30%–60% CHCl_3_ in hexane). Compound **2** was obtained as a yellow oil (509 mg, 0.95 mmol, 86%). ^1^H-NMR (300 MHz, CDCl_3_) δ: 7.85 (2H, d, *J* = 9.3 Hz, aromatic), 7.83 (2H, s, aromatic), 7.13 (2H, d, *J* = 9.3 Hz, aromatic), 4.16 (2H, s, -CH_2__benzylic_); 3.44 (4H, t, *J* = 7.8 Hz, CO-CH_2_-), 2.97 (4H, *J* = 7.0 Hz, -CH_2_Br), 1.92, 1.78, 1.54 (16H, m, -CH_2_-). ^13^C-NMR (75 MHz, CDCl_3_) δ: 198.3 (-CO-); 154.7, 132.7, 132.7, 129.5, 119.9, 116.7 (aromatic); 33.5(-CH _benzylic_); 38.0, 32.6, 27.8, 27.4, 23.4 (-CH_2_-). HRMS *m/z* calc. C_25_H_28_Br_2_O_3_ [M+Na]^+^ 559.3128, found [M+Na]^+^ 559.3126.

#### 3.3.1. General Procedure for Nucleophilic Substitution

Product **2** was dissolved in THF (5–15 mL) and treated with an excess of the amine (5 mmol) at 0 °C, then the solution was stirred overnight at room temperature. After completion (TLC 10% MeOH in DCM), the solvent was evaporated *in vacuo*. The crude product was dissolved in DCM (75 mL), washed three times with saturated aqueous NaHCO_3_ solution (50 mL), dried over Na_2_SO_4_, filtered and taken to dryness *in vacuo*. The crude obtained product was purified by chromatography column (5%–40% MeOH in DCM).

*6-Dimethylamino-1-[7-(6-dimethylaminohexanoyl)-9H-xanthen-2-yl]-hexan-1-one* (**3a**): Compound **2** (200 mg, 0.37 mmol) dissolved in THF (5 mL) and DMA in THF (2 M solution, 1.20 mL) gave **3a** as a white solid (159 mg, 0.343 mmol, 96%); ^1^H-NMR (300 MHz, CDCl_3_) δ: 7.84 (2H, d, *J* = 9.3 Hz, aromatic); 7.83 (2H, s, aromatic); 7.10 (2H, d, *J* = 9.3 Hz, aromatic); 4.15 (2H, s, CH_2__benzylic_); 3.43 (4H, t, *J* = 7.5 Hz); 2.97 (4H, t, *J* = 8.5 Hz); 2.46 (12H, s, -NCH_3_-); 1.92 (4H, m, -CH_2_-); 1.77 (4H, m, -CH_2_-); 1.59 (4H, m, -CH_2_-). ^13^C-NMR (75 MHz, CDCl_3_) δ: 198.5 (-CO-), 155.0, 132.7, 129.9, 128.50, 120.2 116.95 (aromatic), 57.0, 37.3, (NCH_2_), 53.4 (-NCH_3_), 45.3 (COCH_2_-), 37.7 (CH_2__benzylic_), 27.5, 26.4, 23.4 (-CH_2_-). HRMS *m/z* calc. C_29_H_41_N_2_O_3_ [M+Na]^+^ 465.6531, found [M+Na]^+^ 465.6522.

*6-Piperidin-1-yl-1-[7-(6-piperidin-1-yl-hexanoyl)-9H-xanthen-2-yl]-hexan-1-one* (**3b**): Compound **2** (300 mg, 0.53 mmol) dissolved in THF (5 mL) and piperidine (0.7 mL) gave **3b** as a white solid (280 mg, 0.541 mmol, 94%); ^1^H-NMR (300 MHz, CDCl_3_) δ: 7.83 (2H, d, *J* = 9.3 Hz, aromatic); 7.80 (2H, s, aromatic); 7.10 (2H, d, *J* = 9.3 Hz, aromatic); 4.13 (2H, s, CH_2__benzylic_); 2.93 (4H, t, *J* = 7.5 Hz, COCH_2_-); 2.32 (12H, m-NCH_2_); 1.74 (4H, m, -CH_2_-), 1.56 (14H, m, -CH_2_-), 1.39 (6H, m, -CH_2_-). ^13^C-NMR (75 MHz, CDCl_3_) δ: 198.6 (-CO-), 155.1, 132.7, 132.7, 130.1, 120.2, 117.0 (aromatic), 53.9, 53.2, (-NCH_2_-), 37.8 (CO-CH_2_), 27.6 (CH_2__benzylic_), 26.5, 23.5, 23.4, 22.7, 22.7, 22.2 (-CH_2_-). HRMS *m/z* calc. C_35_H_49_N_2_O_3_ [M+Na]^+^ 545.7865, found [M+Na]^+^ 545.7870.

*6-(4-Methylpiperazin-1-yl)-1-{7-[6-(4-methylpiperazin-1-yl)-hexanoyl]-9H-xanthen-2-yl}-hexan-1-one* (**3c**): Compound **2** (200 mg, 0.37 mmol) dissolved in THF (5 mL) and 1-Methylpiperazine (0.5 mL) gave **3c** as a white solid (190 g, 0.33 mg, 93%) ^1^H-NMR (300 MHz, CDCl_3_) δ: 7.83 (2H, d, *J* = 9.3 Hz, aromatic); 7.82 (2H, s, aromatic); 7.09 (2H, d, *J* = 9.3 Hz, aromatic); 4.08 (2H, s, CH_2__benzylic_); 2.93 (4H, t, *J* = 6.8 Hz, -CH_2_CH_2_N); 2.52 (12H, b, -NCH_2_CH_2_N-); 2.30 (6H, s, -NCH_3_); 1.74, 1.56, 1.38 (20H, m, -CH_2_-). ^13^C-NMR (75 MHz, CDCl_3_) δ: 198.9 (-CO-), 155.9, 132.9, 129.8, 128.6, 120.1, 116.9 (aromatic), 60.4, 58.1 (-NCH_3_), 58.0, (N-CH_2_), 53.2 (N-CH_2_CH_2_-N), 46.1, (‑CH_2_N-), 38.4 (CO-CH_2_), 27.6 (CH_2__benzylic_), 26.4, 26.8, 24.4 (-CH_2_-). HRMS *m/z* calc. C_35_H_51_N_4_O_3_ [M+Na]^+^ 575.8156, found [M+Na]^+^ 575.8153.

#### 3.3.2. General Procedure for Xanthene Oxidation

(A) Jones’ reagent preparation: in a 50 mL beaker, Cr_2_O_3_ (7.0 g) was dissolved at 0 °C in water (10 mL) and H_2_SO_4_ (6.1 mL) then additional water (20 mL) was added. (B) The product **3** was dissolved in acetone (5–15 mL) in a two neck flask at 0 °C. An excess of Jones’ reagent (approximately 1 mL, each 10 mmol, solubilized in acetone with a ratio 1:5) was added slowly over 20 min. The reaction mixture was then allowed to warm to room temperature. After 5 h the solution was concentrated and the excess of Jones’ reagent was destroyed with a 5% solution of thiosulfate. The product was extracted three times with DCM, finally the organic layers were washed with saturated aqueous NaCl solution, and dried in Na_2_SO_4_. (TLC 10% MeOH in CDCl_3_), filtered and taken to dryness *in vacuo*. The crude obtained product was purified by flash column chromatography (5%–40% MeOH in CHCl_3_).

*2,7-Bis-(6-dimethylaminohexanoyl)-xanthen-9-one* (**4a**): Compound **3a** (150 mg, 0.32 mmol) was dissolved in acetone (3 mL), then Jones’ reagent (0.5 mL) was added, to give **4a** as a white solid (153 mg, 0.31 mmol, 95%). ^1^H-NMR (300 MHz, CDCl_3_) δ: 8.83 (2H, d, *J_0_* = 2.0 Hz, aromatic); 8.34 (2H, dd, *J*_0_ = 2.0 Hz, *J*_1_ = 8.7 Hz, aromatic); 7.55 (2H, d, *J_1_* = 8.7 Hz, aromatic); 3.05 (4H, t, *J* = 7.7 Hz, -COCH_2_-); 2.38 (4H, t, *J* = 7.10 Hz, -CH_2_N); 2.57 (12H, t, -NCH_3_); 1.75 (4H, m, -CH_2_-); 1.53 (4H, m, -CH_2_-); 1.39 (4H, m, -CH_2_-). ^13^C-NMR (75 MHz, CDCl_3_) δ: 198.5 (Ph-(CO)Ph), 176.4 (Ph-(*CO*)CH_2_-); 158.7, 134.6, 133.6, 127.8, 121.3, 119.1 (aromatic), 8.3, 45.8 (-CH_2_N-), 38.6 (COCH_2_-), 27.3, 26.6, 24.1 (-CH_2_-). HRMS *m/z* calc. C_35_H_47_N_2_O_4_ [M+Na]^+^ 479.6422, found [M+Na]^+^ 479.6420.

*2,7-Bis-(6-piperidin-1-yl-hexanoyl)-xanthen-9-one* (**4b**): Compound **3b** (150 mg, 0.27 mmol) was dissolved in acetone (3 mL), then Jones’ reagent (0.5 mL) was added, to give **4b** as a white solid (143 mg, 0.25 mmol, 93%). ^1^H-NMR (300 MHz, CDCl_3_) δ: 8.88 (2H, s, *J_0_* = 2.1 Hz, aromatic); 8.39 (2H, dd, *J*_0_ = 2.1 Hz, *J*_1_ = 9.0 Hz, aromatic); 7.61 (2H, d, *J_1_* = 9.0 Hz, aromatic); 3.11(4H, t, *J* = 7.5 Hz, -COCH_2_-); 2.66 (8H, m, N-CH_2_CH_2_), 2.57 (4H, t, *J* = 7.0 Hz -CH_2_N); 1.86–1.68 (14H, m, -CH_2_-); 1.51–1.41(8H, m, -CH_2_-). ^13^C-NMR (75 MHz, CDCl_3_) δ: 198.2 (Ph-(CO)Ph), 176.2 (Ph-(*CO*)CH_2_-), 158.4, 134.3, 133.3, 127.5, 121.0, 118.8 (aromatic), 58.9, 54.3 (-CH_2_N-), 38.3 (CO)CH_2_-), 27.2, 26.2, 25.4, 24.0, 23.8 (-CH_2_-). HRMS *m/z* calc. C_35_H_47_N_2_O_4_ [M+Na]^+^ 559.7736, found [M+Na]^+^ 559.7733.

*2,7-Bis-[6-(4-methylpiperazin-1-yl)-hexanoyl]-xanthen-9-one* **(4c**): Compound **3c** (100 mg, 0.17 mmol) was dissolved in acetone (2 mL), then Jones’ reagent (0.4 mL), was added, to give **4c** as a white solid (95 mg, 0.16 mmol, 93%). ^1^H-NMR (300 MHz, CDCl_3_) δ: 8.89 (2H, d, *J_0_* = 2.1 Hz, aromatic); 8.41 (2H, dd, *J*_0_ = 2.1 Hz, *J*_1_ = 9 Hz, aromatic), 7.62 (2H, d, *J_1_* = 9 Hz, aromatic); 3.10 (4H, t, *J* = 8 Hz, -COCH_2_-); 2.43–2.38 (20H, m, -NCH_2_-; 2.35 (6H, s, N-CH_3_); 1.81; 1.61; 1.33 (12H, m, -CH_2_-). ^13^C-NMR (75 MHz, CDCl_3_) δ: 198.3 (Ph-(CO)Ph), 177.9 (Ph-(*CO*)CH_2_-), 158.5, 134.4, 133.5, 127.5, 121.1, 118.9 (aromatic), 58.1 (-CH_2_N), 54.3 (-NCH_2_), 52.6 (-NCH_2_), 38.4 (COCH_2_-), 27.1, 26.4, 26.4, 23.9 (-CH_2_-). HRMS *m/z* calc. C_35_H_47_N_2_O_4_ [M+Na]^+^ 588.8082, found [M+Na]^+^ 558.8080.

*3,6-Bis-(Hydroxy)-xanthen-9-one* (**6**): In a steel-box tube, a 50 mL flask containing 2,2',4,4'- tetrahydroxybenzophenone (5, 509 mg, 2.07 mmol) suspended in water (15 mL) was placed,. The box was heated under magnetic stirring up to 210 °C, so that after 30 min the pressure inside was 35 atm. After 4 h the solution was taken at room temperature and then the mixture was filtered under vacuum with a Gooch funnel. The product was dried under vacuum to give compound **6** as a brown powder (468 mg, 2.05 mmol, 99%). ^1^H-NMR (300 MHz, NaOD) δ: 7.49 (2H, d, *J*_0_ = 9.0 Hz, aromatic); 7.49 (2H, dd, *J*_0_ = 9.0 Hz, *J*_1_ = 1.9 Hz aromatic); 6.12 (2H, d, *J*_1_ = 1.9 Hz, aromatic). ^13^C-NMR (75 MHz, NaOD) δ: 177.0 (Ph-(CO)Ph), 175.4, 159.5, 127.0, 118.8, 109.3 (aromatic). HRMS *m/z* calc. C_13_H_8_O_4_ [M-H]^−^ 249.2180, found [M-H]^−^ 249.2178.

*3,6-Bis-(4-Iodobutoxy)-xanthen-9-one* (**7**): In a 50 mL flask compound **6** (100 mg, 0.43 mmol) and dry K_2_CO_3_ (80 mg, 0.50 mmol) were added to anhydrous DMF (3 mL). When the product was completely dissolved, an excess of 1,4-diiodobutane (0.5 mL) was added at room temperature. To the crude product distilled water (10 mL) was added and the solution was extracted three times with diethyl ether (30 mL). Then, the organic phase was washed three times with saturated aqueous NaCl solution (10 mL) and finally dried over Na_2_SO_4_. The product was purified by column chromatography (30%–60% CHCl_3_ in hexane), to give compound **7** was obtained as a white solid (237 mg, 0.39 mmol, 91%). ^1^H-NMR (300 MHz, CDCl_3_) δ: 8.18 (2H, d, *J*_0_ = 9.0 Hz, aromatic); 6.87 (2H, dd, *J*_0_ = 8.7 Hz, *J*_1_ = 2.1 Hz, aromatic); 6.77 (2H, d, *J*_1_ = 2.1 Hz, aromatic); 4.07 (4H, t, *J* = 6.0 Hz, CH_2_O); 3.27 (4H, d, *J* = 6.6 Hz, -CH_2_Cl); 2.02 (8H, m, -CH_2_-). ^13^C-NMR (75 MHz, CDCl_3_): δ 175.3 (PhCOPh), 163.8, 157.8, 128.1, 115.8, 113.0, 100.7 (aromatic), 67.2(-CH_2_O-), ,29.9, 29.8 (-CH_2_-), 29.9 (-CH_2_I). HRMS *m/z* calc. C_21_H_22_I_2_O_4_ [M+Na]^+^ 615.2228, found [M+Na]^+^ 615.2224.

*3,6-Bis-(4-Dimethylaminobutoxy)-xanthen-9-one* (**8a**): The product was obtained and purified by the same procedure used for compound **2**. Compound **7** (0.3 g, 0.51 mmol) was dissolved in THF (6 mL), then DMA solution (2 M) in THF (1.20 mL) was added, to give **8a** as a white solid (209 mg, 0.49 mmol, 97%); ^1^H-NMR (300 MHz, CDCl_3_): δ 8.18 (2H, d, *J_1_* = 8.7 Hz, aromatic); 6.88 (2H, dd, *J*_1_ = 8.7 Hz and *J*_0_ = 2.1 Hz, aromatic); 6.82 (2H, d, *J_0_* = 2.1 Hz, aromatic); 4.71 (4H, t, *J* = 6 Hz, -OCH_2_-); 2.44 (4H, t, *J* = 4.0 Hz, N-CH_2_-); 2.27 (12H, s, NCH_3_); 1.85 (4H, m, -CH_2_-); 1.68 (4H, m, -CH_2_-). ^13^C-NMR (75 MHz, CDCl_3_) δ: 175.4 (-CO-), 164.1, 157.9, 128.0, 115.7, 113.1, 100.7 (aromatic), 68.3 (-OCH_2_-), 59.1 (CH_2_N), 45.2 (-NCH_3_-), 26.8, 23.9 (-CH_2_-). HRMS *m/z* calc. C_25_H_34_N_2_O_4_ [M+Na]^+^ 449.2281, found [M+Na]^+^ 449.2277.

*3,6-Bis-(4-piperidin-1-yl-butoxy)-xanthen-9-one* (**8b**): The product was obtained and purified by the same procedure as used for compound **2**. Compound **7** (0.3 g, 0.51 mmol) was dissolved in THF (6 mL), then piperidine (0.7 mL) was added, to give **8b** as a white solid (241 mg, 0.47 mmol, 94%); ^1^H-NMR (300 MHz, CDCl_3_) δ: 8.20 (2H, d, *J_1_* = 8.7 Hz, aromatic); 6.90 (2H, dd, *J*_1_ = 8.7 Hz and *J*_0_ = 2.1 Hz, aromatic); 6.83 (2H, d, *J_0_* = 2.1 Hz, aromatic); 4.09 (4H, t, *J* = 6 Hz, -OCH_2_-); 2.38 (4H, t, *J* = 4.0 Hz, N-CH_2_-); 2.38 (12H, m, -NCH_2_-); 1.84 (4H, m, -CH_2_-); 1.71 (4H, m,-CH_2_-); 1.44 (4H, m, -CH_2_-). ^13^C-NMR (75 MHz, CDCl_3_) δ: 175.4 (-CO-), 164.1, 157.9, 128.0, 115.7, 113.1, 100.7, (aromatic), 68.3 (-OCH_2_-), 59.1 (-CH_2_N-), 45.2 (-NCH_2_-), 26.8, 23.9(-CH_2_-). HRMS *m/z* calc. C_31_H_42_N_2_O_4_ [M+Na]^+^ 529.8275, found [M+Na]^+^ 529.8269.

*3,6-Bis-[4-(4-Methylpiperazin-1-yl)-butoxy]-xanthen-9-one* (**8c**): The product was obtained and purified by the same procedure as used for compound **2**. Compound **7** (100 mg, 0.17 mmol) was dissolved in THF (6 mL), then methylpiperazine (0.3 mL) was added, to give **8c** as a white solid (80 mg, 0.15 mmol, 91%); ^1^H-NMR (300 MHz, CDCl_3_) δ: 8.23 (2H, d, *J_1_* = 8.6 Hz, aromatic); 6.94 (2H, dd, *J*_1_ = 8.6 Hz and *J*_0_ = 2.2 Hz, aromatic); 6.79 (2H, d, *J_0_* = 2.2 Hz, aromatic); 4.09 (4H, t, *J* = 6 Hz, -OCH_2_-); 2.38 (4H, t, *J* = 4.0 Hz, N-CH_2_-); 2.38 (20H, m, -NCH_2_-); 2.27 (6H, s, NCH_3_); 1.88 (4H, m, -CH_2_-); 1.69 (4H, m, -CH_2_-). ^13^C-NMR (75 MHz, CDCl_3_) δ: 175.9 (-CO-), 163.2, 158.1, 128.5, 116.0, 113.6, 101.0 (aromatic), 68.3 (-OCH_2_-), 59.9, 59.1, 58.6 (-CH_2_N-), 45.8 (-NCH_3_-), 26.8, 23.9 (-CH_2_-). HRMS *m/z* calc. C_31_H_44_N_4_O_4_ [M+Na]^+^ 559.7276, found [M+Na]^+^ 559.7269.

*9-(4-Iodobutyl)-9H-xanthene* (**9**): To a mixture of xanthene (**1**, 100 mg, 0.54 mmol), previously solubilised in dry THF (5 mL) a solution of BuLi (3 mmol) was added under Ar at room temperature. The simultaneous carbanion formation caused an immediate colour change of the solution from colourless to red. The solution was transferred dropwise to a flask containing 1,4-diidodoproane (0.15 mL, 2 mmol) in dry THF (5 mL) at −50 °C. The reaction was allowed to warm to room temperature after 2 h and the reaction stirred overnight at room temperature. After approx. 14 h (TLC 40% hexane-CHCl_3_ 7:3), the solvent was evaporated *in vacuo*. The crude product was dissolved in DCM (75 mL), washed three times with water (30 mL), dried (Na_2_SO_4_) and taken to dryness *in vacuo*. The crude product obtained was purified by flash column chromatography (0%–40% CHCl_3_ in hexane). A white powder of **9** was obtained (37.7 mg, 0.25 mmol, 42%). ^1^H-NMR (300 MHz, CDCl_3_) δ: 7.27–7.04 (8H, m, aromatic); 4.01 (1H, t *J* = 4 Hz, CH _benzylic_); 3.28 (2H, t, *J* = 3.3 Hz, -CH_2_Br); 1.80 (4H, m, -CH_2_-CH_2_); 1.33 (2H, m, CH-CH_2_). ^13^C-NMR (75 MHz, CDCl_3_) δ: 152.4, 128.7, 127.7, 125.4, 123.3, 116.5 (aromatic), 39.9 (-*CH*-), 39.0, 33.4, 32.9, 24.3 (-CH_2_-). HRMS *m/z* calc. C_17_H_17_OBr [M+Na]^+^ 340.2281, found [M+Na]^+^340.2277.

*6-Bromo-1-[7-(6-bromohexanoyl)-9-(4-iodobutyl)-9H-xanthen-2-yl]-hexan-1-one* (**10**): The product was obtained and purified by the same procedure as used for compound **1**. 9 mg (0.027 mmol) of **9** were used and a yellow oil was obtained (**10**) (16.9 mg 0.022 mmol 84%). ^1^H-NMR (300 MHz, CDCl_3_) δ: 7.88–7.85 (4H, m, aromatic); 7.16 (2H, m, aromatic); 4.13 (1H, m, CH _benzylic_); 3.40 (6H, m, -CH_2_-); 2.37 (4H, m, -CH_2_-); 1.90–1.22 (18H, m,-CH_2_-). ^13^C-NMR (75 MHz, CDCl_3_) δ: 198.7 (-*CO*-), 179.8, 133.1, 129.3, 128.6, 124.9, 116.8 (aromatic), 40.0, 38.5, 38.2, 34.2, 33.7, 33.5, 32.4, 27.7, 23.9, 23.5 (-CH_2_-). HRMS *m/z* calc. C_29_H_35_O_3_Br_3_ [M+Na]^+^ 640.3033, found [M+Na]^+^ 640.3039.

*6-Dimethylamino-1-[9-(4-dimethylaminobutyl)-7-(6-dimethylaminohexanoyl)-9H-xanthen-2-yl]-hexan-1-one* (**11**): the product was obtained and purified by the same procedure as used for compound **2**. Compound **10** (15 mg, 0.21 mmol) was used and **11** was obtained as a white powder (12.2 mg, 0.18 mmol, 90%). ^1^H-NMR (300 MHz, CDCl_3_) δ: 7.88–7.83 (4H, m, aromatic); 7.13 (2H, m aromatic); 4.16 (1H, t *J* = 4.17 Hz, -CH _benzylic_); 2.99 (4H, t, *J* = 3.00 Hz, -CH_2_-); 2.70 (6H, t, *J* = 2.70 Hz, -CH_2_-N-); 2.55 (12H, s, -N(CH_3_)_2_); 2.48 (6H, s, -N(CH_3_)_2_); 1.80–1.14 (18H, m, -CH_2_-). ^13^C-NMR (75 MHz, CDCl_3_) δ: 198.6 (-CO-), 155.0, 132.9, 129.3, 128.4, 124.6, 116.7 (aromatic), 58.5, 58.2 (COCH_2_), 43.9, 43.7 (-N(CH_2_)_2_, 40.3 (-NCH_2_-), 38.2 (*-*CH _benzylic_), 58.4, 58.2, 43.9, 43.7, 40.3, 38.2, 37.9, 25.5, 25.4, 23.6, 22.4 (-CH_2_-). HRMS *m/z* calc. C_29_H_35_O_3_Br_3_ [M+Na]^+^ 564.8124, found [M+Na]^+^ 564.8130.

*1,3-Bis-[(9H-Xanthen-9-yl)]-propane* (**12**). In a 25 mL flask, xanthene (**1**, 350 mg, 1.92 mmol), dissolved in dry THF (10 mL) and diiodopropane (0.2 mL 1.74 mmol) were stirred under argon at 0 °C. n-Butyllithium (1 mL, solution 2.5 M in hexanes) was added dropwise, after which the reaction was heated up to room temperature. After approx 2 h (TLC 40% hexane-CHCl_3_ 7:3), solvent was evaporated *in vacuo*. The crude product was dissolved in DCM (75 mL), washed three times with water (30 mL), dried over Na_2_SO_4_ and taken to dryness *in vacuo*. The crude product was purified by flash column chromatography (0%–40% CHCl_3_ in hexane), to give **12** as a white powder, (410 mg, 0.93 mmol, 48% yield). ^1^H-NMR (300 MHz, CDCl_3_) δ: 7.26–6.98 (16H, m, aromatic); 3.86 (2H, t, -CH _benzylic_); 1.62 (4H, m, -CH_2_-); 1.19 (2H, m, -CH_2_-). ^13^C-NMR (75 MHz, CDCl_3_) δ: 152.4, 128.7, 127.5, 125.8, 123.2, 116.4 (aromatic), 43.9, 43.7 (-N(CH_2_)_2_), 40.3 (-CH _benzylic_); 39.0, 21.9 (-CH_2_-). HRMS *m/z* calc. C_29_H_35_O_3_Br_3_ [M+Na]^+^ 427.5124, found [M+Na]^+^ 427.5110.

*1,3-Bis[6-Bromo-1-[7-(6-bromohexanoyl)-9H-xanthen-2-yl]]-propane* (**13**): The product was obtained and purified by the same procedure used for compound **1***.* Compound **12** (9 mg, 0.017 mmol), AlCl_3_ (0.2 mg) in anhydrous DCM (2 mL) and 6-bromohexanoyl chloride (0.1 mL) of were added at −0 °C under argon. **13** was obtained as a yellow oil, (16.9 mg, 0.015 mmol, 84% yield). ^1^H-NMR (300 MHz, CDCl_3_) δ: 7.81 (4H, d, aromatic); 7.72 (4H, s, aromatic); 7.08 (4H, d, aromatic); 3.98 (2H,t, CH _benzylic_); 3.44 (8H, m -CH_2_-); 2.96 (8H, t, -CH_2_-); 2.38 (4H, t, -CH_2_-); 1.96–1.56 (26H, m, -CH_2_-). ^13^C-NMR (75 MHz, CDCl_3_) δ: 198.5 (-CO-), 155.1, 133.0, 129.2, 128.4, 124.8, 116.7 (aromatic), 40.6 (-CH_2_-), 38.7 (CH _benzylic_), 33.8, 33.5, 32.7, 28.0, 23.5, 21.1 (-CH_2_-). HRMS *m/z* calc. C_53_H_60_Br_2_O_6_ [M+Na]^+^ 1135.6788, found [M+Na]^+^ 1135.6779.

*1,3-Bis[6-Piperidin-1-yl-hexanoyl)-9H-xanthen-2-yl] ]-propane* (**14**): The product was obtained and purified by the same procedure as used for compound **2***.* Compound **13** (16.9 mg, 0.015 mmol) and DMA (0.3 mL, 2M solution in THF) were used. Thirteen mg of **14** (0.011 mmol, 76%) was obtained. ^1^H-NMR (300 MHz, CDCl_3_) δ: 7.80 (4H, dd, aromatic); 7.72 (4H, s, aromatic); 7.08 (4H, d, aromatic); 3.97 (2H, t, CH _benzylic_); 2.93 (8H, t, -CH_2_-); 2.49–2.28 (28H, m -CH_2_-); 1.78–1.24 (50H, m, CH_2_). ^13^C-NMR (75 MHz, CDCl_3_): δ 198.5 (-CO-), 155.1, 133.0, 129.2, 128.4, 124.8, 116.7 (aromatic), 58.7, 54.2, 38.2 (-CH_2_-), 33.1 (CH _benzylic_); 29.7, 27.2, 25.7, 24.9, 24.1, 23.8 (-CH_2_-). HRMS *m/z* calc. C_53_H_60_Br_2_O_6_ [M+Na]^+^ 1135.6788, found [M+Na]^+^ 1135.6779.

### 3.4. Analysis of the DNA-Drug Interactions by ESI-MS

*Instrumentation:* All the experiments were performed on a Q-TOF MICRO spectrometer (Micromass, now Waters, Manchester, UK) equipped with an ESI source, in the negative ionization mode. The rate of sample infusion into the mass spectrometer was 5 or 10 μL/min and the capillary voltage was set to −2.6 kV. The source temperature was adjusted to 70 °C and the source pressure was set at 1.30 mbar. The cone voltage was set to 30 V and the collision energy to 10 V. Full scan MS spectra were recorded in the *m/z* range between 800 and 2,500, with 100 acquisitions per spectrum. Data were analyzed using the MassLynx software developed by Waters.

*Sample preparation protocol*: Oligonucleotides were dissolved in bi-distilled water to obtain the starting stock solutions and were annealed in 150 mM ammonium acetate buffer by heating at 90 °C for 10 min and then cooling slowly to room temperature. The final concentration of oligonucleotides stocks was 50 μM in either duplex or quadruplex units. Ammonium acetate was chosen as the buffer main component for its good compatibility with ESI-MS. Calf thymus DNA (CT) was dissolved in bi-distilled water. Since its average chain length is 13 kb, it was subjected to sonication (Sonyprep 150 sonicator) for 8 min to obtain an average length of 500 bp (according to gel electrophoresis analysis with Mass Ruler DNA ladder mix-low range). Drug stock solutions were prepared by dissolving in bi-distilled water the desired amount of drug hydrochlorides to obtain a final concentration of 100 μM.

Samples were prepared by mixing appropriate volumes of 150 mM ammonium acetate buffer, 50 μM annealed oligonucleotide stock solution, xanthene or xanthone derivatives 100 μM stock solutions and methanol. The final concentration of DNA in each sample was 5 μM (in duplex or quadruplex unit) and the final volume of the sample was 50 μL. Drugs were added at different drug/DNA ratios, ranging between 0.5 and 4. Methanol was added to the mixture just before injection (in a percentage of 15% vol.) after the complexation equilibrium in ammonium acetate was established, in order to obtain a stable electrospray signal. As a reference, samples containing only 5 μM DNA with no drug were prepared in each series.

Samples for competition experiments were prepared following the procedure described above, adding an appropriate volume of CT solution. Final concentrations of quadruplex DNA and drug solutions were always 5 μM and CT was added at two different duplex/quadruplex ratios (1 and 5), calculated on the basis of the phosphate group concentrations. In order to minimize casual errors each experiment has been repeated at least three times, in the same experimental conditions, and data were processed and averaged with the SIGMA-PLOT software.

Binding constants (K_1_ and K_2_) and percentage of bound DNA have been calculated according to previously reported formulae [39]. Considering drug-DNA complexes in 1:1 and 2:1 stoichiometry, which have been proven to be the main species present in solution in all the experiments, the formation of such complexes can be represented by two distinct equilibria:

DNA + drug 1:1

drug + 1:1 2:1

which are in turn described by the following two equations:

K_1_ = [1:1]/([DNA] [drug]) (1)

K_2_ = [2:1]/([1:1] [drug]) (2)

where [DNA], [drug], [1:1] and [2:1] represent respectively the concentrations of the different species in solution: DNA (duplex or quadruplex depending on the oligonucleotide used), the ligand, the 1:1 and 2:1 drug-DNA complexes at equilibrium. The association constants K_1_ and K_2_ (Equations (1) and (2), respectively) can be calculated directly from the relative intensities of the corresponding peaks found in the mass spectra, with the assumption that the response factors of the oligonucleotides alone and of the drug-DNA complexes are the same, so that the relative intensities in the spectrum are proportional to the relative concentrations in the injected solution:

(DNA)/[DNA] = I(1:1)/[1:1] = I(2:1)/[2:1]
(3)

In this way, since DNA and drugs initial concentrations (C0 and C0’ respectively) are known, it is possible to obtain the concentration of each species appearing in 1 and 2:

[j] = C0 • I(j)/(I(DNA) + I(1:1) + I(2:1))
(4)

[drug] = C0’ – [1:1] – 2[2:1]
(5)

where [j] stands for [DNA], [1:1] or [2:1].

The constants were determined at different drug/DNA ratios, depending on the intensity of the signals 2.5:5, 5:5, 7.5:5, 10:5 and 20:5 micromolar concentrations ratios. A further manipulation of the data leads to the calculation of the amount of ligand bound, according to an equation developed by de Pauw and his group [24] derived from Equation (4):

[ligand bound] = C_0_ (I(1:1) + 2I(2:1))/(I(DNA) + I(1:1) + I(2:1)) (6)

This parameter, representing the total amount of the drug bound to DNA, is useful to compare the efficiency of different ligands in DNA binding, when they interact as single molecules. Since planar aromatic derivatives are known to also interact with DNA in self-aggregate forms, we decided to carry out a slightly different approach, according to another equation, described by Brodbelt and co-workers [41] which has been demonstrated to be more correct in such cases and was specifically applied to the study of the interactions between DNA and the derivatives:

% bound DNA (%b) = 100 • (I(1:1) + I(2:1) )/(I(DNA) + I(1:1) + I(2:1) )
(7)

This parameter (%b) represents the percentage of DNA bound to the ligand.

### 3.5. Analysis of the DNA-Drug Interactions by FRET

#### 3.5.1. Sequence Preparation

All oligonucleotides were purchased from Eurogentec (Seraing, Belgium) and used without anyfurther purification. Analyses were performed on the following oligonucleotides:

HTelo-21: 5’-FAM-GGGTTAGGGTTAGGGTTAGGG-TAMRA-3’
c-kit-1: 5’-FAM-AGAGGGAGGGCGCTGGGAGGAGGGGCT-TAMRA-3’
bcl2: 5’-FAM-5'-GGGCGCGGGAGGAAGGGGGCGGG-3'-TAMRA-3’
T loop: 5’-FAM-TATAGCTATA-HEG-TATAGCTATA-TAMRA-3’

where HEG is a hexaethyleneglycol linker [(-CH2-CH2-O-)_6_] to make the hairpin loop, TAMRA (6-carboxytetramethylrhodamine) is the acceptor fluorophore, FAM (6-carboxyfluorescein) is the donor. The sequences were stored at −20 °C as 20 μM stock solutions in water. The oligonucleotides were annealed as 400 nM (2×) stock solutions in FRET buffer (potassium cacodylate 60 mM, pH = 7.4; cacodylic acid purchased from Sigma, Gillingham, UK), heating at 85 °C for 10 min, then slowly cooling to room temperature. The final concentration of the DNA in the FRET plate was 200 nM.

#### 3.5.2. Sample Preparation and Measurement

The drugs were stored at 4 °C as 10 mM stock solutions in DMSO. The original stocks were first diluted with a 1 mM aqueous solution of HCl to 1 mM concentration, and the further dilutions were performed in FRET buffer, in order to obtain 2× stock solutions of the final concentrations. The experiments were performed in 96-well plates (MJ Research, Waltham, MA, USA). Briefly, each well was loaded with 50 μL of the 400 nM DNA solution, together with either 50 μL of the 2× stock solutions of the drug in FRET buffer (final volume per well = 100 μL), or 50 μL of FRET buffer for the blank. All the measurements were taken on a DNA Engine Opticon (MJ Research) with excitation at 450–495 nm and detection at 515–545 nm. Fluorescence readings were taken at 0.5 °C intervals over the range 30–100 °C; a constant temperature was maintained for 30 s prior to each reading. All the experiments were performed in triplicate [44].

## 4. Conclusions

Four different xanthene and xanthone series are introduced here as new G-quadruplex interactive compounds. Molecular modeling studies especially docking, have elucidated what may be the characteristics needed to improve each series of new compounds. The different series of G-quadruplex binding ligands has been synthesized in a small number of steps, each using a different synthetic approach. All the compounds synthesized contain an aromatic core and several side chains, in order to establish the best parameters for interaction with G-quadruplex. ESI-MS assays appear to be promising as a rapid method to evaluate G-quadruplex binding and selectivity with respect to duplex DNA.

The results show that different molecular features contribute to the efficiency of G-quadruplex interactive compounds in binding and stabilizing different G-quadruplex structures. In particular, when the π-π interactions are the same in a series of homologous compounds, such as the xanthene or the xanthone derivatives of the first three series, the length and basicity of the side chains play a major role in modulating the behavior of the different compounds, as previously reported for several other classes of compounds [4,5,10].

The most interesting ligand for targeting telomeric quadruplex DNA is the trisubstuted compound XA3c (**14**). This ligand showed high binding affinity by mass spectral assays to the telomeric G-quadruplex in potassium solution, as confirmed by FRET experiments. This compound merits further biological studies as a potential anti-telomerase agent. Moreover, the same molecule could be exploited as a stabilizing agent of G-quadruplex-based aptamers, in line with previous studies carried out by our and other groups [46,47,48].
